# Quantifying coronary sinus flow and global LV perfusion at 3T

**DOI:** 10.1186/1471-2342-9-9

**Published:** 2009-06-11

**Authors:** Karin Markenroth Bloch, Marcus Carlsson, Håkan Arheden, Freddy Ståhlberg

**Affiliations:** 1 Philips Medical Systems, Best, the Netherlands; 2 MR department, Lund University Hospital, Lund, Sweden; 3 Department of Clinical Physiology, Lund University Hospital, Lund, Sweden; 4 Department of Medical Radiation Physics, Lund University, Lund, Sweden; 5 Department of Radiology, Lund University Hospital, Lund, Sweden

## Abstract

**Background:**

Despite the large availability of 3T MR scanners and the potential of high field imaging, this technical platform has yet to prove its usefulness in the cardiac MR setting, where 1.5T remains the established standard. Global perfusion of the left ventricle, as well as the coronary flow reserve (CFR), can provide relevant diagnostic information, and MR measurements of these parameters may benefit from increased field strength. Quantitative flow measurements in the coronary sinus (CS) provide one method to investigate these parameters. However, the ability of newly developed faster MR sequences to measure coronary flow during a breath-hold at 3T has not been evaluated.

**Methods:**

The aim of this work was to measure CS flow using segmented phase contrast MR (PC MR) on a clinical 3T MR scanner. Parallel imaging was employed to reduce the total acquisition time. Global LV perfusion was calculated by dividing CS flow with left ventricular (LV) mass. The repeatability of the method was investigated by measuring the flow three times in each of the twelve volunteers. Phantom experiments were performed to investigate potential error sources.

**Results:**

The average CS flow was determined to 88 ± 33 ml/min and the deduced LV perfusion was 0.60 ± 0.22 ml/min·g, in agreement with published values. The repeatability (1-error) of the three repeated measurements in each subject was on average 84%.

**Conclusion:**

This work demonstrates that the combination of high field strength (3T), parallel imaging and segmented gradient echo sequences allow for quantification of the CS flow and global perfusion within a breath-hold.

## Background

Access to data on global LV perfusion and CFR can aid in the diagnosis of coronary disease. As 95% of the LV perfusion drains to the right atrium through the coronary sinus [1], flow in this vessel is a good representation of the global LV perfusion. The feasibility of measuring CS flow has been demonstrated in previous studies performed at 1.5T, using phantoms [2] and animal models [3]. The latter study showed good agreement between the CS flow determined by PC MR and that registered with an invasive flow meter. Comparisons between CS flow obtained by PC MR and PET have shown good correlation between the methods [4-6]. From CS flow and LV mass, the CFR can be assessed, if measurements are performed both in rest and stress [4-7].

Phase contrast MR (PC MR) has been used for clinical investigations for well over a decade [8]. Segmented PC sequences [9] allow for breath-hold scans that reduce respiratory artefacts. However, their increased acquisition windows decrease time resolution and can introduce blurring. Parallel imaging techniques, such as Sensitivity Encoding (SENSE) [10], can help in regaining some of this loss, as a shorter acquisition time can be traded for a shorter echo train, still keeping the scan time within a breath-hold. The accuracy, reproducibility and noise behaviour of PC MR images acquired with SENSE, have been investigated. From these data, it was concluded that PC MR results are not compromised by the use of relatively low reduction factors [11,12].

Obtaining measurement at 3T rather than 1.5T could reduce some of the difficulties associated with CS flow quantification. The increased SNR allows for better spatial resolution, and also translates into better velocity-to-noise (VNR) ratio [13]. PC MR studies at 3T are sparse [14] and concentrated to 3D-studies [15-17]. The combined utility of 3T for improved signal-to noise and parallel imaging for reduction of acquisition time has not yet been evaluated for flow measurements in the coronary vessels. Hence, this study aimed to execute an improved CS flow and LV perfusion protocol by combining 3T with SENSE and k-space segmentation, and to evaluate it in phantom studies as well as in vivo.

## Methods

All scans were performed on a Philips Intera 3.0T MR scanner, equipped with a 6-channel cardiac coil, and scans were retrospectively triggered using vector ECG.

### Phantom experiments

For testing the phase-versus-flow linearity and the influence of motion on the flow measurements, a phantom study with two different phantoms was performed prior to the volunteer studies. A flow-sensitized segmented gradient echo sequence (TFE) with SENSE = 2.0 was used for PC imaging. The flow sensitive data and the reference data were acquired in an interleaved fashion. The in-plane spatial resolution was 1.3 × 1.3 mm^2^, and a slice thickness of 8 mm was used. Further sequence parameters are given in Table 1.

**Table 1:** Sequence parameters

Parameter	bTFE	PC MR
TR	4 ms	5.4 ms *
TE	2 ms	3.5 ms *
α°	40°	10°
BW	940 Hz/pixel	680 Hz/pixel *
SENSE reduction	2.0	2.0
In-plane resolution	1.3 × 1.3 mm^2^	1.3 × 1.3 mm^2^*
Slice thickness	8 mm	8 mm
Number of slices	1^a^, 15–20^b^	1
Heart phases	30	21
Segmentation factor	9	10
v_enc_	-----	56 cm/s *
Time resolution	35 ms	45 ms *
Acquisition time	10 s/slice	22 s *

Table 1 summarizes the sequence parameters for the balanced TFE sequence and for the PC MR sequence. The former was used for LV-imaging and positioning of the PC MR, and the latter for flow measurements in the CS. Varying parameters are given as the average of the values used for the different scans, and marked with *.

^a^2- 3- and 4-chamber views. ^b^Short axis stack, covering the left ventricle and left atrium

Phantom 1 was comprised of a cylinder, 15 cm long and 7 cm in diameter, filled with distilled water. Two inner tubes with a diameter of 4.8 mm, and wall thickness <0.1 mm, were positioned inside the phantom, parallel to the long axis. The tubes were connected by a hose, and flow in the two tubes was identical in magnitude but opposite in direction. Gravitation-driven flow through the tubes was maintained between one elevated water container and one container at ground level. Seven different flow values, from 0 ml/s to 7 ml/s, were used. The flow sequence was repeated three times for each selected flow value. The flow determined with PC MR was compared with manually measured flow using a timer-and-beaker setup. This consisted of a beaker that was filled with roughly 2 litres of the flowing water. The time it took to fill the vessel was measured using a stopwatch. The precise volume of the water in the container was determined by weighing the vessel.

Phantom 2 was used to investigate effects of in-and through-plane motion, and was comprised of a cylindrical dish phantom, approximately 8 cm in diameter, filled with Ni-doped agarose gel. The T_1 _and T_2 _of the gel were comparable to those of myocardium at 3T (T_1 _= 867 ms and T_2 _= 57 ms [18]). One tube of the same type as in Phantom 1 was imbedded in the gel. The phantom setup allowed for phantom motion in-plane, through-plane or a combination of the two. The motion of the phantom was used to trigger the sequence. The complete setup of the phantom experiment is described in [2]. The maximum motion of the phantom was approximately two vessel diameters between consecutive time frames. In comparison, the maximum motion of the CS in vivo was found to be on the order of 1 vessel diameter/time frame. The average pulsatile flow in the phantom tube was 5 ml/s, with a peak value of 8 ml/s and a minimum value of 2 ml/s.

### Volunteer experiments

**Figure 1:**
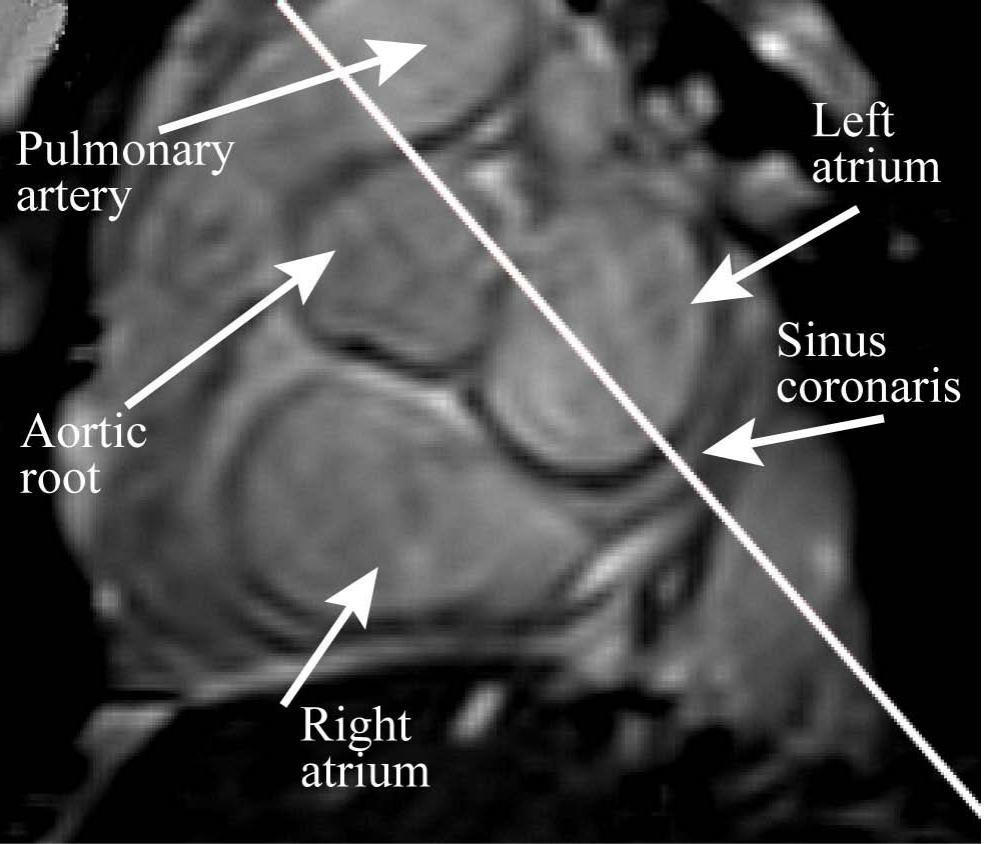
**Positioning of the flow measurement**. In the short-axis slice shown, the coronary sinus is clearly visible, and the slice for flow measurement (white line) is placed perpendicular to the vessel.

Twelve healthy volunteers (1 female, mean age 33 ± 9 years, range 22 to 51 years), with no history of cardiac events, were included in the study. A balanced, segmented gradient echo sequence (bTFE) with SENSE = 2.0 was used for the cine morphological imaging. The slice thickness was 8 mm, without gap between adjacent slices, and the true in-plane resolution was on average 1.3 × 1.3 mm^2^. The sequence parameters are given in Table 1. Single-slice images were acquired in the 2-, 3-, and 4-chamber views. In the short axis (SA) orientation, a stack of 16–20 slices was acquired to cover the complete LV and the left atrium. All images were obtained during breath-hold. The coronary sinus was identified on the basal slices of the SA stack, and the plane for flow measurement was prescribed perpendicular to the direction of flow in the vessel (Figure 1). The complete protocol lasted 40–50 minutes, depending on the time used for planning of the CS flow measurements. The study was performed under local ethical committee approval and informed consent was given by each volunteer.

The PC MR sequence described under Phantom Experiments was also used for the CS flow measurements, see Table 1. The velocity encoding (v_enc_) was optimized for each volunteer by analyzing a line profile through the SC on a PC-image taken with v_enc _= 70 cm/s. In eight cases, v_enc _= 50 cm/s was appropriate, in the other cases v_enc _= 70 cm/s was retained. The true in-plane resolution was on average 1.3 × 1.3 mm^2^, ranging from 1.2 × 1.2 mm^2 ^to 1.5 × 1.5 mm^2^. Twenty-one cardiac phases were acquired in 19–24 seconds, depending on heart rate, corresponding to a temporal resolution of 35–55 ms. The flow measurement was repeated three times in each volunteer.

### Evaluation

In phantoms as well as in vivo, delineations of ROIs (Regions-Of-Interest) were made during simultaneous viewing of magnitude and phase images. Continuous variables are presented as mean ± SD. Agreement between results from two independent observers was expressed as mean difference ± SD, and the limits of agreement are shown in a Bland-Altman plot as mean difference ± 2SD.

In the phantoms, a simple background correction was done by subtracting the average phase of two ROIs placed in the gel background, close to the tubes, from the measured value inside the tube. The reason for not using the phase correction algorithm supplied by the manufacturer was to investigate the phase background present in the scanner. The influence of ROI size on the measured flow was previously investigated by Arheden et al. [2]. Repeating that analysis for our sequence, we selected eleven circular ROIs positioned at the centre of the phantom tube, and analysed the flow calculated from these ROIs. The eleven ROIs had diameters of 1–10 mm (1 mm increments) and 15 mm. By angulating the slice with respect to the flow direction from -10° to 10°, in increments of 2.5°, the effect of angulation could be considered. This effect was also previously considered by Arheden et al. [2]. The ROI was selected as a circle with the same area as the nominal tube area, as were the ROIs in that evaluation of a moving phantom [2].

The phase background as well as the measurement situation is expected to be more complicated in vivo than in the phantom setup. To get good results for CS flow, the in vivo flow data were phase corrected with algorithms supplied by the manufacturer. These include analytical correction of Maxwell effects [19] to the second order, and a fit to slowly varying phase background to remove linear as well as non-linear phase variations induced for example by eddy currents. No manual phase subtraction was performed in this case. The LV-mass evaluation as well as CS delineation for one experiment in each volunteer was made by two independent observers (KMB, MC). The CS delineation of all repeated CS flow experiments was made by one observer (KMB). Data were analyzed using the vendor supplied cardiac package (ViewForum, v4.1) as well as the freeware analysis package Segment http://segment.heiberg.se[20]. LV delineation was semi-automatic in Segment, and manual in ViewForum. CS delineation was manual in both cases. The flow in the CS was obtained as the product of the mean velocity in the ROI delineating the CS at each cardiac phase and the area of this ROI. The flow curve was integrated over the RR-interval to provide the average CS_flow_. In the literature, LV perfusion is usually presented as average flow per minute per g, and to conform to this, the CS_flow _was multiplied with the heart rate. Left ventricular perfusion (LV_perf_) was obtained by dividing CS_flow _with the left ventricular mass (LV_mass_). The inter-observer variability was calculated as the mean difference ± SD of the results of the two observers.

## Results

### Phantom experiments

**Figure 2:**
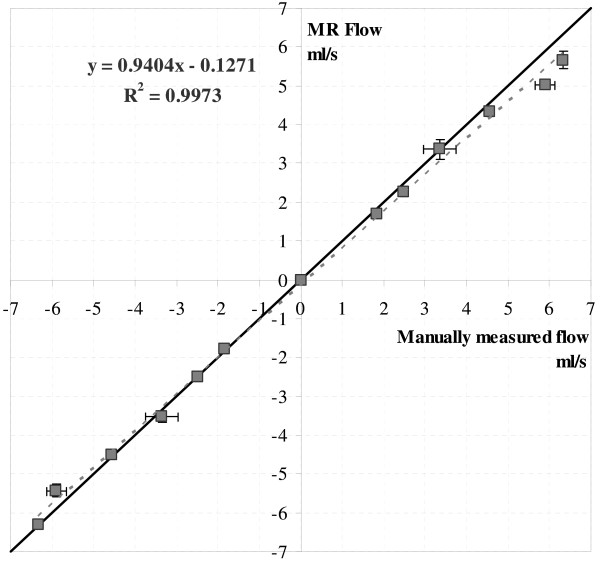
**Linearity of phase signal versus nominal flow**. Flow measured by the segmented PC MR sequence plotted against the manually measured flow (timer and beaker) shows a linear relation. The dashed line shows a linear fit of the data, while the solid line shows the line of identity.

The correlation between the flow determined by MR and by timer and beaker is shown in Figure 2. As seen from this figure, the expected linear relation between flows measured with the both methods was obtained with a R^2 ^value of 0.997. The uncertainty in the manual measurements was 3 ± 4%, gauged by the discrepancy between repeated measurements. As expected, [2], the flow obtained from the ROI analysis matched the manually measured flow when the ROI area equalled the nominal tube area. The maximum deviation of the measured flow for an angulated slice from that at 0° was 5%.

The phantom experiments showed that both in- and through-plane motion affect the shape of the flow curve. The through-plane motion did not change the integrated flow over one period of motion. For through-plane motion, the underestimation of velocity in some phases is cancelled by the overestimation in other phases. However, to observe the true flow profile, correction of the through-plane motion is vital [21].

In-plane motion resulted in the integrated flow being underestimated by between 20% and 30%, which is in the same order of magnitude as theoretical results [22]. Errors due to in-plane motion arise both from delineation difficulties and induced phase errors [22]. In our phantom studies, we observed a large motion blurring in certain frames, making delineation very difficult.

### Volunteer experiments

**Figure 3:**
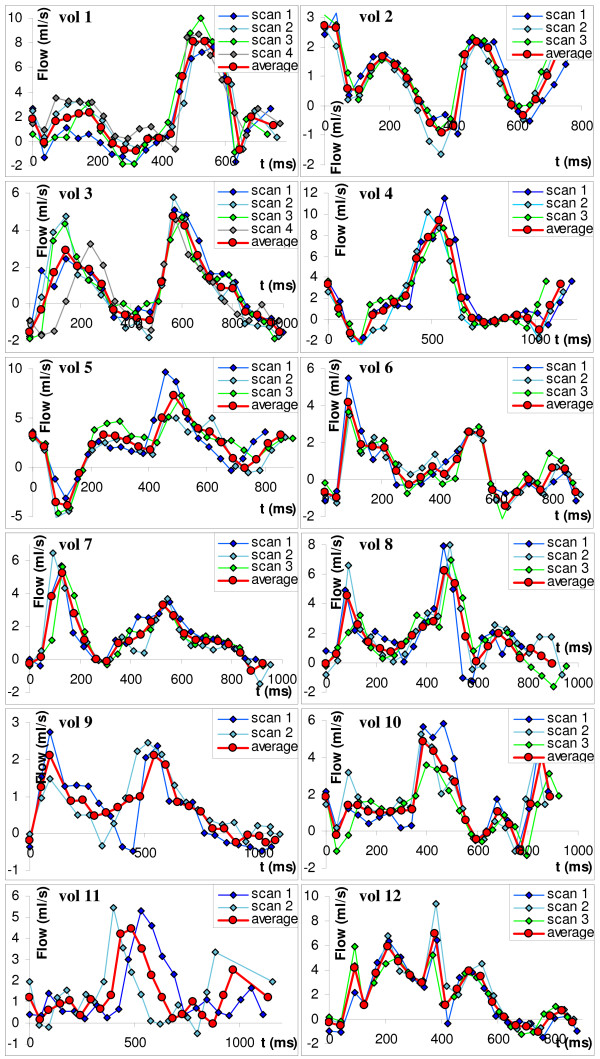
**The flow in the coronary sinus for all volunteers**. Flow in the coronary sinus for volunteers 1–12, measured in three consecutive experiments (for vols. 1 and 3, there were four consecutive experiments). The blue, light blue and green diamonds denote the first, second and third measurements, respectively, while red circles show the calculated mean of the three measurements. The images show the results obtained by the first observer. In two cases (volunteers 9 and 11), one scan is excluded due to breathing artefacts.

All 12 volunteers were scanned successfully. Two flow measurements (in two different volunteers) were excluded due to respiratory artefacts. The flow curves for all volunteers are shown in Figure 3. As seen from this figure, the CS flow curves show biphasic behaviour, in accordance with literature [23,24]. The physiological variation between subjects is large, although the general curve shape is similar. The first flow peak was seen around 0.2·RR and the second around 0.6·RR, where RR is the time interval between two consecutive R-peaks.

**Figure 4:**
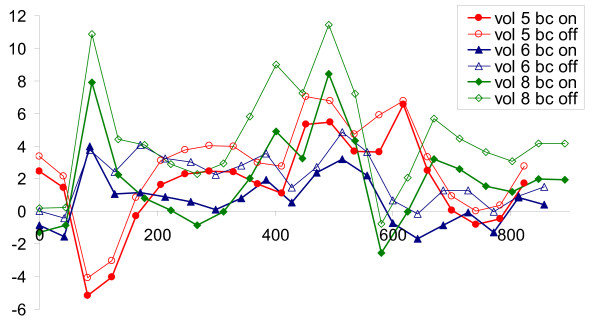
**Comparison of flow curves with and after without phase correction**. Data from three volunteers (nr 5, 6 and 8) were analyzed before as well as after phase background correction. This figure shows the resulting flow curves. The data from the three volunteers are shown by red circles, blue triangles and green diamonds, respectively. Filled symbols denote data after background phase correction, and the empty symbols are data before background correction. Note that all cases were analytically corrected for Maxwell effects. It is clear that the background correction reduces phase, and thus reduces the flow compared to the uncorrected data.

The data from three volunteers was reconstructed with and without background phase correction, using the correction for Maxwell effects in all reconstructions. When not applying the background correction, the integrated flow was heavily overestimated. The background phase mainly arises from eddy current induced phase errors and through-plane motion. Figure 4 shows these three flow curves before and after phase correction for, and Table 2 gives the mean integrated flow before and after phase correction.

**Table 2:** CS_flow _with and without phase background correction

CS_flow _(ml/min)	vol. 5	vol. 6	vol. 8
before phase correction	165	122	271
after phase correction	95.4	37.1	116

Table 2 presents the CS_flow _(ml/min) for the three volunteers where data was analyzed before as well as after correction for background phase. The CS_flow _is heavily overestimated if background phase correction is not performed.

**Figure 5:**
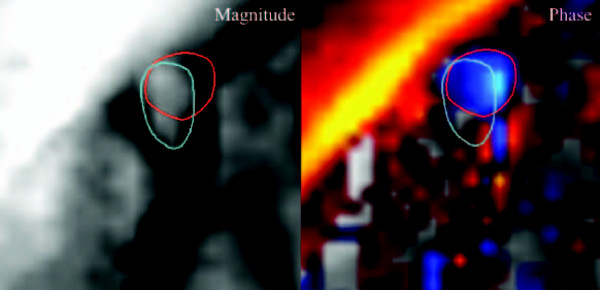
**Mismatch between modulus and phase images**. An example of mismatch of the CS position in the modulus (left) and phase (right) images. The red circle shows the CS position in the phase image and the blue circle shows the apparent CS position in the magnitude image. The image is taken in late diastole, when the CS moves rapidly upwards. Blue color denotes flow into the page, while red color shows flow up from the page. The example is taken from vol. 8.

In-plane motion between the spatial and velocity encoding gradients result in a mismatch between the position of the CS on the modulus image and corresponding phase image (Figure 5). In this work, all twelve volunteers showed mismatch in at least one cardiac phase. At most, half of the heart phases were affected, and on average 7–8 heart phases had a modulus-phase mismatch. The magnitude of the error was in no case larger than 2 pixels, 0.5 pixels being the most common value. The position mismatch and its correlation to the flow curve are individual. The mismatch usually occurs in phases just before or coinciding with phases with maximum flow.

The ROI delineation was done with simultaneous inspection of modulus and phase images. In frames with position mismatch, the ROI was drawn on the phase image. Using only the modulus images for delineation changed the integrated flow by up to 40%. The flow profiles were similar, but the peak values were underestimated if only the modulus information was used when delineating the CS.

**Figure 6:**
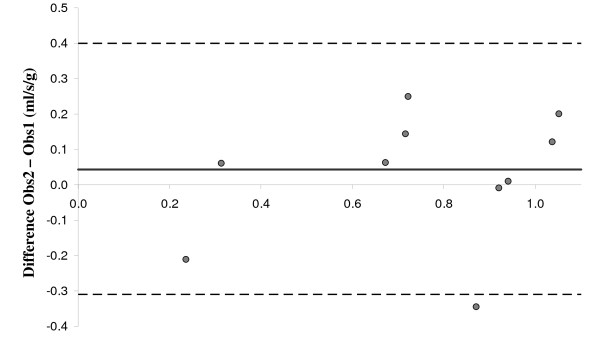
**Inter-observer variability in the perfusion data**. Bland-Altman plot showing the inter-observer variability in the LV perfusion data. The full drawn line shows the bias, and the dotted lines show the ± 2SD interval. The variability originates solely difference in CS_flow_, as the same LV mass was used for both observers.

The results of flow and perfusion measurements for all volunteers are presented in Table 3, where the values given are averages over the three scans for each subject. The variability, defined as (1SD/average) in each volunteer, was on average 16% (range 5–29%). The average CS flow and global LV perfusion for the group were 88 ± 33 ml/min and 0.60 ± 0.22 ml/min·g, respectively, as measured by observer 1. The inter-observer variation was deduced from the data evaluated by both observers, was found to be 7.40 ± 31.22 ml/min for CS_flow_, and 0.043 ± 0.181 ml/min·g for LV_perf_. This is illustrated in the Bland-Altman plot in Figure 6.

**Table 3:** Summary of the results for all volunteers

	Sex	CS_flow _Av ± 1SD (ml/min)	LV_mass _(g)	LV Perf. (ml/min·g)
vol. 1	M	132 ± 32	194	0.68
vol. 2	M	55 ± 10	181	0.31
vol. 3	M	54 ± 16	183	0.30
vol. 4	M	110 ± 20	147	0.75
vol. 5	M	126 ± 26	147	0.85
vol. 6	M	38 ± 1.8	130	0.30
vol. 7	F	86 ± 9.2	75	1.15
vol. 8	M	109 ± 13	126	0.86
vol. 9	M	43 ± 3.7	133	0.32
vol. 10	M	87 ± 17	148	0.59
vol. 11	M	90 ± 6.7	147	0.61
vol. 12	M	124 ± 17	152	0.82

Average		88 ± 33	147 ± 31	0.60 ± 0.22

The result for each volunteer is the mean value of three measurements, evaluated by one observer. The error of the CS flow measurement, defined as (1SD/average) in each volunteer, was on average 16% (range 5–29%).

## Discussion

**Figure 7:**
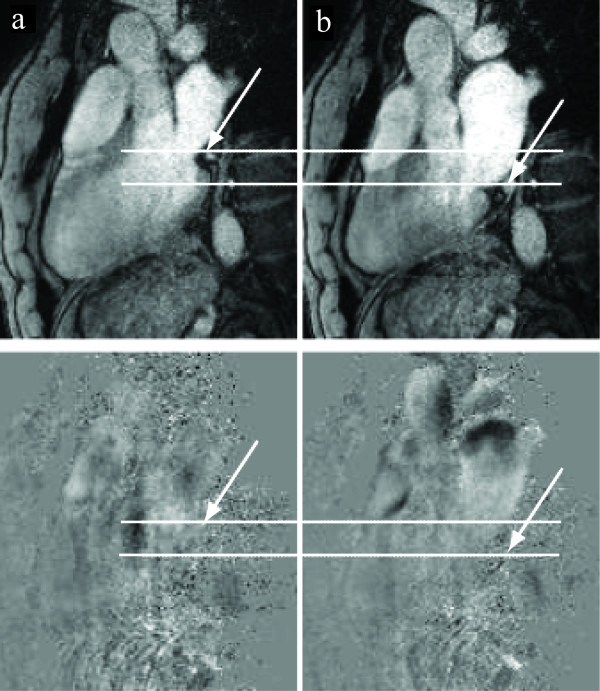
**Variation in position and size of the CS**. Example of the variation in size and position of the coronary sinus during the cardiac cycle. The CS (arrow) is shown here in modulus (top row) and phase images (bottom row) in diastole (column a) and systole (column b), for one volunteer (vol.6).

Flow quantification in the coronary arteries is hampered by physiological as well as technical limitations. The size of the CA demands an in-plane resolution on the order of 1 mm^2^, limiting the available MR-signal, while partial volume effects still affect the data. Furthermore, insufficient time resolution will result in blurring. The winding anatomy of the CA, in combination with prominent motion during the cardiac cycle, makes it difficult to find an imaging plane that remains perpendicular to the vessel during the cardiac cycle. Despite these obstacles, several groups have undertaken the task of flow measurements in the CA [25-29]. The measurements have been validated against Doppler flow [30], PET [31], and animal models [32]. The cross sectional area of the CS is larger than the cross-sectional area of the CA, making less demands on spatial resolution. One study shows that size of the CS in normal subjects varies between 8.3 ± 2.5 mm at onset of the P-wave and 4.8 ± 1.9 mm at maximum LV contraction [33]. On the other hand, the CS exhibits a larger variation in area during the cardiac cycle than the coronary arteries, and it also is displaced on the order of several vessel diameters over each RR-interval (see Figure 7 and [4]). However, the present study shows that quantification of CS flow and global LV perfusion at 3T is feasible, and that results are reproducible if known error sources are taken into account.

### Positioning

The positioning is most easily done on basal short axis cine slices, where the CS is seen as it joins the right atrium. The slice should be placed perpendicularly to the vessel close to the ostium, so that all venous branches have joined the coronary sinus, ensuring that the measurement includes all venous flow. On the other hand, the atrium must not move into the imaging slice during the cardiac cycle. In this case, erroneous results will be obtained, as the flow pattern in the atrium is different from what is seen inside the vessel. The flow at the CS orifice differs between individuals. The CS valve is only a fold of endocardium which suppresses retrograde flow [34], and it varies in size. In this work, we did not compare different positions of the measurement slice. As the CS moves during the coronary cycle, it is probable that flow measurements at different cardiac phases will have different angulations of the slice with respect to the vessel. However, the phantom measurements indicate that misangulation is not a major error source for angular deviations below 10°, in agreement with the Wolf et al[35].

### ROI definition

In earlier phantom experiments [2] repeated in this study, it was shown that the best flow estimate is obtained if the ROI has the same area as the lumen of the vessel. In vivo, the true size is not known, and in the case of the CS, it fluctuates over the cardiac cycle. Manual delineation of the vessel is therefore required at each cardiac phase. Furthermore, a slight overestimation of the area has less impact on the obtained flow than an underestimation, under the assumption of static background [2].

A combined view of both magnitude and phase images showed to be essential when delineating the CS, for several reasons. The magnitude images in some cases cannot separate the CS from the neighbouring left circumflex coronary artery. On the phase images, two distinct flow patters are in these cases seen within the single bright spot on the magnitude image. In other cases, the CS is hypointense on the magnitude image, while the phase image shows the true location of the vessel. In the same way, bright fat signal can be mistaken for bright blood signal on the modulus images. During the cardiac cycle, the CS is displaced several vessel diameters (see Figure 7 and [4]). Displacement artefacts constitutes a second reason for using both phase and magnitude images when delineating the ROI. Due to the time difference between the frequency, phase and velocity encodings, there will be a mismatch in vessel position between magnitude and phase images if the vessel moves, or if the flow is oblique to the slice [36,37]. An example of this mismatch is shown in Figure 5. In all cardiac phases where there was a position mismatch between phase and magnitude images, the delineation was made on the phase image. Only using the modulus image for delineation underestimate the flow, as the in-plane motion and the position mismatch often coincides with peak flow values.

### Partial volume effects

Limited spatial resolution in combination with small, moving vessels can create problems with partial volume effects. It has been shown that covering the lumen with 16 pixels will give an maximal error of the flow measurement of 10% [38]. In our analysis, the resolution was high enough to permit 15–50 pixels over the vessel cross section, depending on the cardiac phase and the individual. The spatial resolution in this work is better than what has been achieved at 1.5T when averaging has not been used. However, a thinner slice than used in this work would further reduce through-plane partial volume effects.

### In- and through-plane motion

Phantom results have suggested that errors from through-plane motion are corrected for using an adequate background phase correction. This was verified in our phantom experiments, and was also commented on by Koskenvuo et al. [6].

In-plane motion during the segment results in phase errors and position mismatch. It is not straight-forward to correct for phase errors produced by in-plane motion, but rather revised encoding strategies are needed to avoid them [22]. In the same paper, [22], it is shown that phase contrast measurements are insensitive to in-plane motion between velocity encodings, but that segmented sequences are sensitive to motion over the segment duration.

It is worth noting that the phantom experiments using the present phantom setup had a larger maximum acceleration than expected in the in vivo case and this part of the phantom investigation hence represents a worst-case scenario. In comparison, the error from in-plane motion was in [2] found to be rather small. To further improve results, it would be useful to investigate corrections for in-plane motion, including alternative encoding schemes.

### Observed outliers

The LV perfusion in four out of the twelve volunteers was only half of the average LV perfusion (see Table 2). Inspection of the individual flow curves for these volunteers, shows that one volunteer (vol. 5) showed a large retrograde flow during the first 200 ms. In one case (vol. 2), the flow pattern was tri-phasic and in the other two cases (vols. 3 and 9), a normal biphasic flow patterns was seen. Examination of the slice placement in the outliers did not reveal any sign of mispositioning relative to the ostium.

### Breath-holding

When measuring a structure as mobile as the CS, reduction of motion artefacts is clearly important. Motion control can be achieved with breath-holding, separation of the scan into two or three breath-holds, or navigator triggering. The first of these options is disadvantageous, as the consecutive breath-holds may not be identical, resulting in slice mispositioning. Navigator triggering is feasible, but prolongs the scan to a point where the time gained by segmenting the sequence is lost. It is also not clear how the CS position correlates with the diaphragm position, making it non-trivial to optimize the gating strategy. Thus, in this study, we chose to use breath-holding for suppression of respiratory motion. It has previously been shown that the velocity in the RCA was decreased by 15–20% in breath-holding as compared to a respiratory gated scan [25]. In previous studies of CS flow, breath-holding [5,6], shallow breathing [7], as well as free breathing [4,23,39] has been used. The choice of method coincides with the sequence type used, as only segmented sequences allow for breath-holding. However, one can argue that short breath-holds without an increase in the intra-thoracic pressure will only have minor influences on the hemodynamics [40].

### Relation to earlier results

In our study, the average CS flow was determined to be 88 ± 33 ml/min. This is in range with what has been found in previous studies [5,4,7,23,39], where values from 69 ml/min [4] to 144 ml/min [23] have been reported. The average value of LV perfusion for the group, 0.60 ± 0.22 ml/min·g, is also in accordance with published values, which lie between 0.53 ± 0.14 ml/min·g and 0.73 ± 0.23 ml/min·g [5,6,4,39]. Previous studies were carried out on 1.5T units (except [23], which used 0.6T). Comparisons between MR and a reference standard, PET, have been carried out by two groups [4,6]. There, it is shown that CS_flow_/LV_mass _obtained by the two methods correlate closely (r = 0.82, p < 0.001 [6] and r = 0.93 [4]). The repeatability and intra-observer variations reported in [4] are within the same range as ours.

Cardiac MR at 3T is often thought of as hampered by artefacts. These are most striking in balanced SSFP-sequences, as the homogeneity of the static magnetic field deteriorates with field strength. In this study, this was relevant for the LV-imaging and images for positioning of the CS. The balanced sequence used (Table 1) was found to yield images without artefacts in relevant areas when appropriate shimming was used. No specific artefacts were seen in the PC images.

The use of 3T allowed for improved spatial resolution due to the increase in SNR. Resolution is of outermost importance when investigating vessels as small as the CS. The in-plane spatial resolution used in this work is better than corresponding works at 1.5T when only using one average. The increased SNR at 3T also translates into an increased velocity-to-noise ratio (VNR) in the acquired PC data. Furthermore, a short acquisition for breath-hold imaging is important to reduce respiratory artefacts, as well as displacement of the CS from the imaging plane. Segmented sequences shorten the acquisition time, so that also a high-resolution scan fits into a breath-hold. Adding SENSE to the protocol decreases the SNR somewhat, but brings a reduction in scan time that can be exchanged for a shorter echo train. A shorter echo train is desirable, as a better temporal resolution reduces blurring. A good temporal resolution captures fast changes in the flow profile better, and decreases the risk of underestimating peak flow. In this work, 21 heart phases could be obtained, also an improvement compared to earlier work. However, a mismatch in the position of the vessel between magnitude and phase images was seen, indicating a need for still better time resolution. Since the study described here was carried out, gradient systems with better performance have become available, and the sequence used in this work could thus be optimized further. Specifically, the acquisition time and the degree of segmentation could be reduced.

## Conclusion

The proposed method for quantifying CS flow and global LV perfusion at 3T with segmented PC MR, accelerated by SENSE, holds promise for clinical use. In future studies, the protocol can be used in rest and stress to determine LV_perf _as well as CFR in patients with coronary disease.

## Competing interests

KMB is employed by Philips Medical Systems. This company is neither financing the study nor the manuscript.

## Authors' contributions

KMB did the sequence optimization, carried out and analyzed the phantom experiments, analyzed the volunteer data, and drafted the manuscript. MC carried out the volunteer scannings, and analyzed part of the volunteer data. HA conceived of the study and participated in its design, as well as gave support to the analysis. FS participated in the design of the study, and gave support in the sequence optimization as well as analysis phases. In addition, all authors read and made substantial contributions to the final manuscript.

## Pre-publication history

The pre-publication history for this paper can be accessed here:

http://www.biomedcentral.com/1471-2342/9/9/prepub
